# Amelioration of Rheumatoid Arthritis by *Fragaria nubicola* (Wild Strawberry) via Attenuation of Inflammatory Mediators in Sprague Dawley Rats

**DOI:** 10.3390/medicina59111917

**Published:** 2023-10-30

**Authors:** Kiran Mashaal, Arham Shabbir, Muhammad Shahzad, Aisha Mobashar, Tasleem Akhtar, Tabinda Fatima, Bushra Riaz, Rana Alharbi, Afreen Fatima, Abdulkareem A. Alanezi, Ashfaq Ahmad

**Affiliations:** 1 Department of Pharmacology, Faculty of Pharmacy, The University of Lahore, Lahore 54000, Pakistan; kiranmashaal@gmail.com (K.M.); aishamobashar@gmail.com (A.M.); 2 Department of Pharmacology, Institute of Pharmacy, Faculty of Pharmaceutical and Allied Health Sciences, Lahore College for Women University, Jail Road, Lahore 54000, Pakistan; 3 Department of Pharmacology, University of Health Sciences, Lahore 54000, Pakistan; shahzad912@hotmail.com (M.S.); a_tasleem89@yahoo.com (T.A.); 4 Department of Pharmaceutical Chemistry, College of Pharmacy, University of Hafr Al Batin, Hafr Al Batin 39524, Saudi Arabia; tabinda@uhb.edu.sa; 5 Department of Pharmacy Practice, College of Pharmacy, University of Hafr Al Batin, Hafr Al Batin 39524, Saudi Arabia; bushrariaz@uhb.edu.sa (B.R.); rsalharbi@uhb.edu.sa (R.A.); afatima@uhb.edu.sa (A.F.); ashfaqa@uhb.edu.sa (A.A.); 6 Department of Pharmaceutics, College of Pharmacy, University of Hafr Al Batin, Hafr Al Batin 39524, Saudi Arabia

**Keywords:** rheumatoid arthritis, inflammation, cytokines, immunopharmacology, medicinal plants

## Abstract

*Background and Objectives: Fragaria nubicola* has never been evaluated scientifically for its anti-arthritic potential despite its use in folkloric systems of medicine. The research was conducted to assess the potential of *F. nubicola* against rheumatoid arthritis. *Materials and Methods:* The current study provided scientific evidence by evaluating the effects of plants using an in vivo CFA-induced model of arthritic rats and subsequent microscopic histopathological evaluation of ankle joints along with the determination of paw edema using a digital water displacement plethysmometer. The study also gave insight by determining levels of pro-inflammatory cytokines, matrix metalloproteinase enzymes (MMPs), prostaglandin E2 (PGE2), nuclear factor kappa B (NF-κB), vascular endothelial growth factor (VEGF), and biochemical and hematological parameters. GCMS analysis was also conducted for the identification of possible anti-inflammatory plant constituents. *Results:* The data showed that *F. nubicola*-treated groups attenuated the progression of arthritis and paw edema. Microscopic histopathological evaluation validated the anti-arthritic potential by showing amelioration of bone erosion, infiltration of inflammatory cells, and pannus formation. RT-PCR analysis displayed that treatment with *F. nubicola* down-regulated IL1β, IL6, TNFα, NF-κB, VEGF, MMP2, MMP3, and MMP9 levels. Moreover, ELISA exhibited a reduction in levels of PGE2 levels in treatment groups. The levels of RBCs, platelets, WBCs, and Hb content were found to be nearly similar to negative control in the treated group. Statistically, a non-significant difference was found when all groups were compared for urea, creatinine, ALT, and AST analysis, indicating the safety of plant extract and fractions at test doses. GCMS analysis of extract and fractions showed the existence of many anti-inflammatory and antioxidant phytochemicals. *Conclusion:* In conclusion, *F. nubicola* possessed anti-arthritic properties that might be attributed to the amelioration of MMPs and pro-inflammatory cytokines.

## 1. Introduction

Rheumatoid arthritis (RA) is termed an autoimmune and chronic inflammatory disease [1]. RA is one of the most common types of chronic inflammatory arthritis with unknown etiology, which happens frequently due to synovial inflammation and tissue damage [2]. The approximate prevalence of RA is 0.5–1% [3]. The early stage of this disorder shows signs of heat, swelling, pain, and decreased joint function, whereas the late stage shows symptoms of joint stiffness and deformity accompanying bone damage and disability risk [4]. 

In the pathogenesis of rheumatoid arthritis, pro-inflammatory cytokines play a dominant role by stimulating articular cartilage hyperplasia [5]. Interleukin (IL)-6, IL-1β, and TNFα (tumor necrosis factor) are the leading pro-inflammatory cytokines that stimulate the destruction of adjacent joint tissue and facilitate the continuation of chronic inflammatory synovitis [6]. TNFα is a well-known cytokine for regulating the production of other pro-inflammatory cytokines in the rheumatoid synovial tissue [7]. PGE2 is also considered a key inflammatory prostaglandin which takes a part in the pathogenesis of RA [8]. The transcription factor, NF-κB, regulates the gene expression in numerous cellular responses, such as inflammation, cell proliferation, apoptosis, and immunity [9]. VEGF creates crosstalk between angiogenesis and joint inflammation in RA by promoting synovial endothelial cell stimulation [10]. MMPs such as MMP-3, MMP-2, and MMP-9 destroy non-collagen matrix components of the joints, and their levels are found to increase in arthritis [11].

Non-steroidal anti-inflammatory drugs (NSAIDs), disease-modifying anti-rheumatic drugs (DMARDs), analgesics, and biological agents are currently in practice for the treatment of RA. NSAIDs successfully reduce symptoms like pain, swelling, and stiffness of joints but are unable to inhibit the advancement of disease [1,12]. Most of the time, using anti-inflammatory drugs is not advisable because these drugs do not stop the progression of the disease and can further cause worsening of some of the side effects. So, there is a strong desire to develop new therapeutic agents with minimum side effects [13]. Herbal medicine has its beginning in ancient civilization and plays an essential part in healthcare. Most of the world’s population uses herbal medicines primarily for their health care and to promote wellness [14]. Medicinal plants are becoming a priority day by day over conventional medicine due to their effectiveness, minimal side effects, and low cost [15].

*Fragaria nubicola* (Lindl. ex Hook.f.) Lacaita is commonly known as wild strawberry and belongs to the Rosaceae family. It is a perennial herbaceous plant that grows horizontally on the ground [16]. It is majorly found in Pakistan, Nepal, Afghanistan, Bhutan, Kashmir, Myanmar, and Sikkim [17]. The 27 genera and 160 species of this plant family are found in Pakistan, while 85 genera and nearly 3000 species are found in Europe and North America [18]. The fruits of this plant have a delightful aroma. The plants generally range in height from 4 to 25 cm. *F. nubicola*, a diploid species, is unique as it has 14 chromosomes, while other species in the same family have only 7. Another distinguishing feature of *F. nubicola* is the presence of sepals on the outer part of its fruits [19]. In the traditional system of medicine, the plant is known for its anti-rheumatic potential [20]. In addition, different parts of this plant in folkloric medicine are used to treat dermal infections, stomach disorders, urinary ailments, and inflammation [17]. Previously conducted Pharmacological studies reported that *F. nubicola* possessed antioxidant, anti-hyperlipidemic [16], cytoprotective [21], and antidepressant activities [18]. Despite its anti-inflammatory potential in folkloric systems of medicine, the scientific data regarding anti-arthritic potential is scarce. 

Based on the plant’s traditional uses against rheumatic disorders, as well as previously reported antioxidant potential, it was hypothesized that the plant might possess ameliorative properties against FCA-induced arthritis. Therefore, the current study aimed to determine the therapeutic potential of methanol (FN-Metha) extract of *F. nubicola* and its n-hexane (FN-Hexa) and ethyl acetate (FN-Ethaceta) fractions for the treatment of rheumatoid arthritis using CFA-induced arthritic rat model.

## 2. Materials and Methods

### 2.1. Plant Used and Collection

The plant was collected in the month of September 2018 from Azad Jammu Kashmir Medicinal and Aromatic Plants Herbarium (AJKMAPH), a project of the Pakistan Agricultural Research Council (PARC). The plant was identified by Dr-Zaheer-ud-din Khan, Distinguished professor, Department of Botany, G.C University, Lahore, and a specimen voucher (GC.Herb.Bot.3543) was submitted in the herbarium. 

### 2.2. Methanol Extract Preparation 

The whole plant was cleaned and later dried under shade. Afterward, the plant was ground to obtain fine powder. Nearly one kilogram of powder was added in about three liters of methanol in a dark glass container at room temperature for 7 days with daily repeated shaking. After 7 days, the material was first passed by muslin fabric and later via a filter sheet (Whatman number 1). The obtained blackish-green filtrate was vaporized under reduced pressure using a rotary evaporator (Lab tech EV3111plus). The percentage yield of semi-solid extract was calculated as 16% [22]. All the solvents used were of analytical grade (Sigma-Aldrich, St. Louis, MO, USA).

### 2.3. Preparation of n-Hexane and Ethyl-Acetate Fractions

The methanol extract was dissolved in 500 mL distilled water. The fractionation was performed in a separating funnel by mixing with 500 mL of n-hexane. The n-hexane fraction was collected using liquid-liquid extraction and was evaporated under reduced pressure using a rotary evaporator. The n-hexane fraction yield was calculated as 4%. After that, the same procedure was repeated to obtain ethyl acetate fraction. The ethyl acetate fraction yield was calculated as 4.3% [23]. 

### 2.4. Animals Housing Conditions

Sprague Dawley rats of both genders, 150–250 g, 6 to 8 weeks old, were used for the activity. Required animals were retained for one week in the animal house of the Faculty of Pharmacy, The University of Lahore, before starting the activity. Animals were provided standard conditions of diet, tap water, 12 h dark/light cycles, temperature (23–27 °C), and humidity (50–60%). The anti-arthritic activity was permitted by the Institutional Research Ethics Committee at the University of Lahore (IREC-2019-91) [24].

### 2.5. Experimental Design

The 36 rats were equally divided into six groups. The doses of the treatment groups were determined using a pilot study. Group-1 (Negative Control): Healthy rats received only normal saline. Group-2 (Arthritic Control): CFA was introduced in healthy rats to produce arthritis, and after that, only normal saline was given to rats [24]. Group-3 (FN-Metha): Arthritic rats received methanol extract orally at a dose of 500 mg/kg body weight for 15 consecutive days. Group-4 (FN-Hexa): Arthritic rats were treated with n-hexane fraction orally at the dose of 500 mg/kg body weight for 15 continuous days. Group-5 (FN-Ethaceta): Arthritic rats were given ethyl acetate fraction orally at a dose of 500 mg/kg body weight for 15 days continuously. Group 6 (Piroxicam): The rats were intraperitoneally injected with piroxicam (10 mg/kg b.w) [22]. The duration of treatment was similar to other plant-treated groups.

### 2.6. Induction of Arthritis

At day 0, arthritis was induced by injecting 0.15 mL CFA (Santa Cruz Biotechnology) in the sub-plantar area of the left hind paw of rats in all groups except healthy rats. On the 8th day of arthritic induction, the treatment was started and continued for the next 15 days. On the 23rd day of treatment, all animals were euthanized [25]. 

### 2.7. Assessment of Arthritic Development

The existence and severity of the disease were checked using a macroscopic arthritic scoring method. The presence of visible signs in the injected paw at days 8, 13, 18, and 23 of arthritic induction was measured using the following standards: 0 was for normal signs; 1 for minor inflammation and swelling; 2 for mild inflammation and swelling; 3 for moderate inflammation and swelling; 4 for severe inflammation and edema [26].

### 2.8. Paw Volume Assessment Using Plethysmometer

A digital water displacement plethysmometer (LE 7500, Panlab) was used to detect the paw volume of both left and right hind paws on the 8th, 13th, 18th, and 23rd day of arthritic induction, where the right paw worked as a negative control. The rise in paw volume was determined by calculating the difference among the paw volumes of the left injected paw and right non-injected paw. Percentage inhibition was calculated using a previously published formula [25].

### 2.9. Determination of Histopathological Parameters

Briefly, on the 23rd day, ankle joints were removed and fixed in 10% formalin. The joints were then soaked in a decalcifying solution. The tissues were treated with ethanol dilutions, and paraffin blocks were prepared. Afterward, blocks were sliced at 5 μm thickness and stained with hematoxylin and eosin (H&E) [27]. The results were semi-quantified for bone erosion, pannus formation, and infiltration of inflammatory cells by a blinded histopathologist using the following scale: 0, normal; 1, minimum; 2, mild; 3, moderate; and 4, severe [28]. 

### 2.10. Determination of PGE2 Levels by ELISA 

For estimation of PGE2 quantity in serum, sandwich ELISA (Enzyme-linked immunosorbent assay) was used according to kit assay procedure (Bioassay technology laboratory). Optical density was observed at 450 nm [29]. 

### 2.11. Determination of mRNA Expression Levels of MMP2, MMP3, MMP9, NF-κB, IL6, IL1β, TNFα, and VEGF 

Blood samples were initially processed with TRIzol reagent (Thermo Scientific, America), followed by processing with chloroform, isopropyl alcohol, and 75% ethanol to extract total RNA, as per standard protocol. A Nanodrop spectrophotometer was used for the quantification of extracted RNA samples. cDNA form quantified RNA was synthesized using kit protocol (Thermo Scientific, Waltham, MA, USA) [30]. PCR product was made by mixing 1 µL of cDNA, 1 µL of forward primer, 1 µL of reverse primer, 2 µL of nuclease-free water, and 5 µL of PCR Master Mix (Thermo Scientific). The PCR products were then processed in a thermal cycler (Veriti 96-well thermal cycler) under specific conditions. Thermal cycler was designed for 39 cycles of denaturation (95 °C for 10 s), annealing (IL6:59 °C, MMP3:54 °C, IL1β: 57 °C, MMP9: 56.1 °C, NF-κB: 58.4 °C, TNFα: 60 °C, VEGF: 60 °C and MMP2: 85 °C for 20 s), and extension (72 °C for 30 s). GAPDH was used as a reference. Primers sequences of MMP2 (Forward:5′- GCAACAAGTATGAGAGCTGC-3′, Reverse: 5′- CGGTCATCATCGTAGTTGGT-3′), MMP3 (Forward: 5′-CCTTTTGATGGGCCTGGAAT -3′, Reverse: 5′-GTGACATCATCTTGTCCATCG -3′), MMP9 (Forward: 5′- CCACCGAGCTATCCACTCAT-3′, Reverse: 5′- GTCCGGTTTCAGCATGTTTT-3′), NF-κB (Forward: 5′-CCGAGATAATGACAGCGTGT -3′, Reverse: 5′-CCTTGGGAACGATATGATGG -3′) and IL-1β (Forward: 5′-CCTGCTAGTGTGGATGTTC -3′, Reverse: 5′-GAGGTGCTGAGTTACCAGTT -3′) were designed manually by Ensemble Genome Browser. Sequences of primers such as IL6 (Forward: 5′-GTCAACTCCATCTGCCCTTCAG-3′, Reverse: 5′-GGCAGTGGCTGTCAACAACAT-3′), TNFα (Forward: 5′-ACAAGGCTGCCCCGACTAT -3′, Reverse: 5′-CTCCTGGTATGAAGTCCGAAATC -3′) [25], and VEGF (Forward: 5′-GTTCAGAGCGGAGAAAGCATT -3′, Reverse: 5′- CTTGCAACGCGAGTCTGTGT-3′) [31] were chosen from previously published article. The PCR product was processed via gel electrophoresis and gel documentation imaging system for visualization, and later densitometry was performed for semi-quantification [32]. 

### 2.12. Biochemical and Hematological Parameters

The blood was collected using the intracardiac puncture technique. Serum was collected from the blood via a centrifugation process (at 5000 rpm for 5 min) to estimate the levels of ALP (Alkaline phosphate), ALT (Alanine aminotransferase), and AST (Aspartate aminotransferase), creatinine, bilirubin, and urea [26]. These levels were determined by using a chemistry analyzer and kit manufacturer’s protocol to analyze any toxic effects of plant extract and fractions on the liver and kidney. The counts of platelets, WBCs, RBCs, and hemoglobin content were also assessed [29].

### 2.13. GC-MS (Gas Chromatography-Mass Spectrometry) Analysis

GC-MS (GCMS-5975c) analysis for plant extract and fractions was performed using previously published methodology with some modifications [33]. The semi-solid extract was mixed with 1 mL of their respective solvent, such as methanol, n-hexane, and ethyl acetate, and then passed through a syringe filter (0.2 µm) to remove any impurities from the liquid. GC-MS analysis was processed with the following conditions: capillary column was HP-5MS (30 m × 250 μm × 0.25 μm film thickness), and Inert gas was helium (99.999%) with a flow rate of 1 mL/min. Initially, oven temperature was operated at 60 °C for 0 min, then at 5 °C /min to 80 °C for 2 min, and then at 10 °C /min to 310 °C for 4 min. The total run time of the extract and fractions solution was 33 min, and the mode was split less. The relative voltage for the mass spectrophotometer was 47, and the quadrupole analyzer was programmed at 200 °C. The mass-to-charge range was set at 30 to 700. 

### 2.14. Statistical Analysis

Graph Pad Prism 5 was applied to evaluate the data. All values were expressed in the form of mean ± standard deviation. One-way ANOVA followed by Tukey’s post hoc test was utilized for the purpose of comparison. 

## 3. Results

### 3.1. F. nubicola Reduced Arthritic Advancement

The disease was not induced in the normal control group, so the values of this group were taken as 0. Continuous growth of arthritis was seen in the arthritic control group because treatment was not given to this group. Prominent reduction in arthritic score was seen in all plant-treated groups, as well as in the piroxicam group, as shown in Table 1. 

### 3.2. F. nubicola Reduced Paw Volume

**Figure 1:**
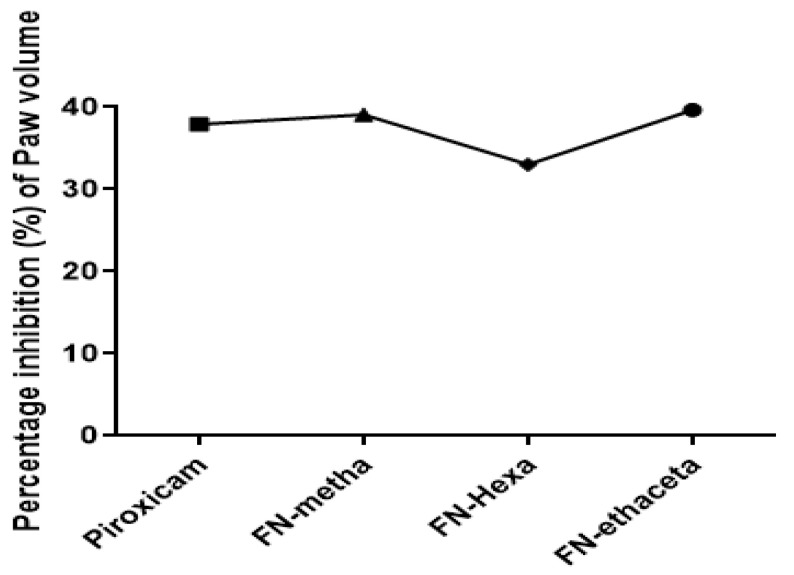
Percentage inhibition of paw volume when compared with the arthritic group. Treatment with ethyl acetate fraction of plant showed the highest inhibition of paw volume.

The disease was not induced in the normal control group, so the values of this group were taken as 0. A notable reduction in paw volume was seen in all plant-treated groups, as well as the piroxicam group, as shown in Table 2. Percentage inhibition of paw volume was also determined using the previously reported formula. Figure 1 showed that Piroxicam (37.93), FN-Metha (39.02%), FN-Hexa (33%), and FN-Ethaceta (39.65%) inhibited paw volume when compared with the arthritic control group.

### 3.3. Microscopic Analysis Showed That F. nubicola Reduced Histopathological Parameters

**Figure 2:**
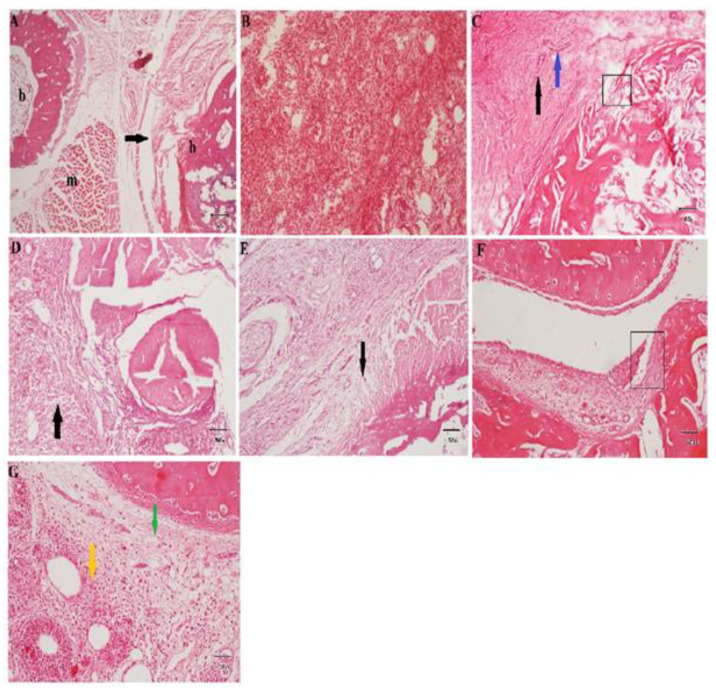
Photomicrographs of histopathological analysis (H&E stained). Normal control ((**A**)× 40): normal bone (b), muscles (m), and synovial layer (black arrow) were observed. Arthritic control: severe infiltration of mononuclear inflammatory cells was noticed ((**B**) × 100). Marked pannus formation (black arrow), cartilage erosion (rectangle), and angiogenesis (blue arrow) ((**C**) × 100) were seen. Piroxicam ((**D**) × 100): mild pannus formation (black arrow) was noticed over the bone. FN-Metha ((**E**) × 100): mild pannus formation (black arrow) was found. FN-Hexa ((**F**) × 100): mild eroded joint cartilage (rectangle) was observed. FN-Ethaceta ((**G**) × 100): moderate infiltration of inflammatory cells (yellow arrow) and fibrosis (green arrow) was seen.

FN-Metha extract, FN-Hexa, and FN-Ethaceta fractions showed a reduction in pannus development, bone erosion, and inflammatory cell infiltration. H&E stained images (Figure 2) showed that all treated groups prominently attenuated the pannus formation, bone erosion, and inflammatory cell infiltration when compared with the arthritic control group (Table 3). 

### 3.4. F. nubicola Significantly Reduced PGE2 Levels

**Figure 3:**
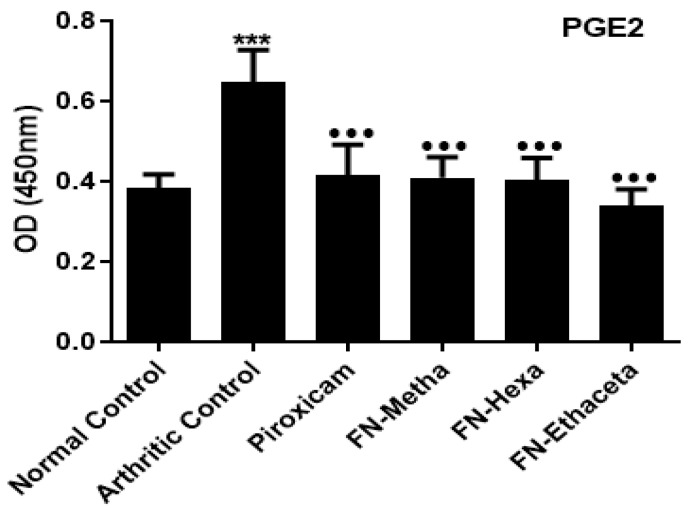
FN-Meth, FN-Hexa, and FN-Ethaceta remarkably decreased the levels of PGE2 as compared to the arthritic control group. Here, ^●●●^ *p*< 0.001 as compared to the arthritic group whereas, *** shows the comparison between the normal control and arthritic group.

PGE2 levels were prominently higher in arthritic control rats (0.646 ± 0.080) as compared to normal control rats (0.383 ± 0.0341). FN-Metha (0.408 ± 0.051), FN-Hexa (0.404 ± 0.053) and FN-Ethaceta (0.340 ± 0.039) significantly reduced the PGE2 levels. Piroxicam also significantly decreased (0.415 ± 0.076) the PE2 Levels as compared to arthritic control (Figure 3).

### 3.5. F. nubicola Significantly Reduced IL1β, IL6, TNF-α, and NF-κB Expression Levels

**Figure 4:**
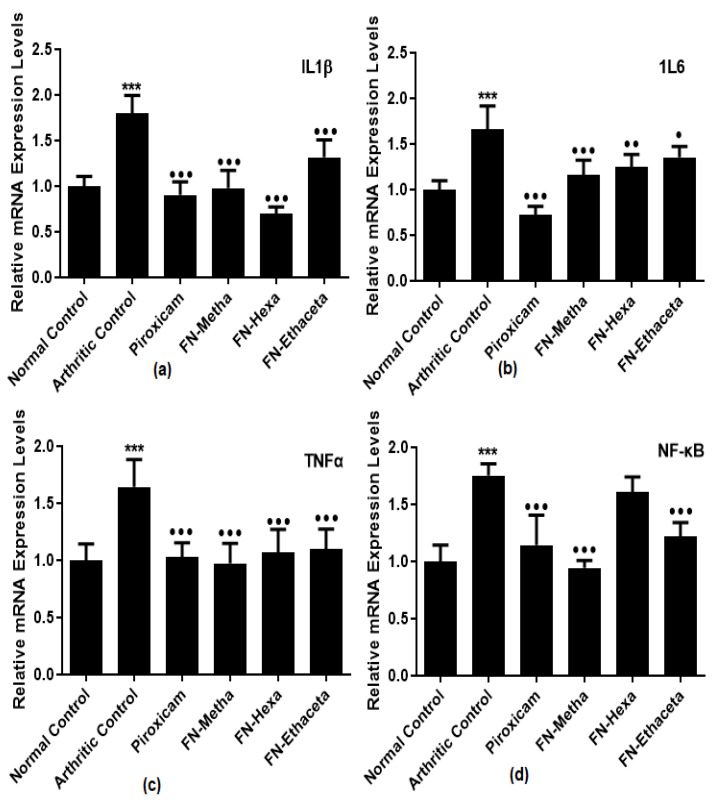
FN-Metha, FN-Hexa, and FN-Ethaceta decreased the expression levels of (**a**) IL1β, (**b**) IL6, and (**c**) TNFα while FN-Metha and FN-Ethaceta decreased the (**d**) NF-κB expression levels as compared to the arthritic control group. Here, ^●●●^ *p*< 0.001, ^●●^ *p*< 0.01, and ^●^ *p*< 0.05 as compared to the arthritic group, whereas *** shows the comparison between the normal control and arthritic group.

IL1β levels were increased in arthritic control rats (1.794 ± 0.201) as compared to normal control rats (1.000 ± 0.106). FN-Metha (0.978 ± 0.193), FN-Hexa (0.695 ± 0.079) and FN-Ethaceta (1.316 ± 0.190) groups displayed significant reduction. Piroxicam also displayed comparable results (0.902 ± 0.145) (Figure 4a). 

Expression levels of IL6 were also elevated in the arthritic control group (1.659 ± 0.259) as compared to the normal control group (1.001 ± 0.095). Treatment with all treated groups, such as FN-Metha (1.159 ± 0.163), FN-Hexa (1.251 ± 0.136), FN-Ethaceta (1.356 ± 0.116), and Piroxicam (0.724 ± 0.092) down-regulated the levels of IL6 as compared to arthritic control group (Figure 4b).

Elevated TNFα expression levels were noticed in the arthritic control group (1.645 ± 0.238) as compared to the normal control group (1.001 ± 0.141). All treated groups such as FN-Metha (0.973 ± 0.175), FN-Hexa (1.069 ± 0.201), FN-Ethaceta (1.069 ± 0.177), and Piroxicam (1.028 ± 0.124) down-regulated the levels of TNFα in comparison with arthritic control group (Figure 4c). 

NF-κB expression levels were significantly increased in the arthritic control group (1.754 ± 0.101) as compared to the normal control group (1.000 ± 0.142). All treated groups such as FN-Metha (0.942 ± 0.065), FN-Hexa (1.609 ± 0.131), FN-Ethaceta (1.217 ± 0.123), and Piroxicam (1.145 ± 0.260) down-regulated the levels of NF-κB in comparison with arthritic control group (Figure 4d). 

### 3.6. F. nubicola Ameliorated Expression Levels of MMP2, MMP3, MMP9 and VEGF

**Figure 5:**
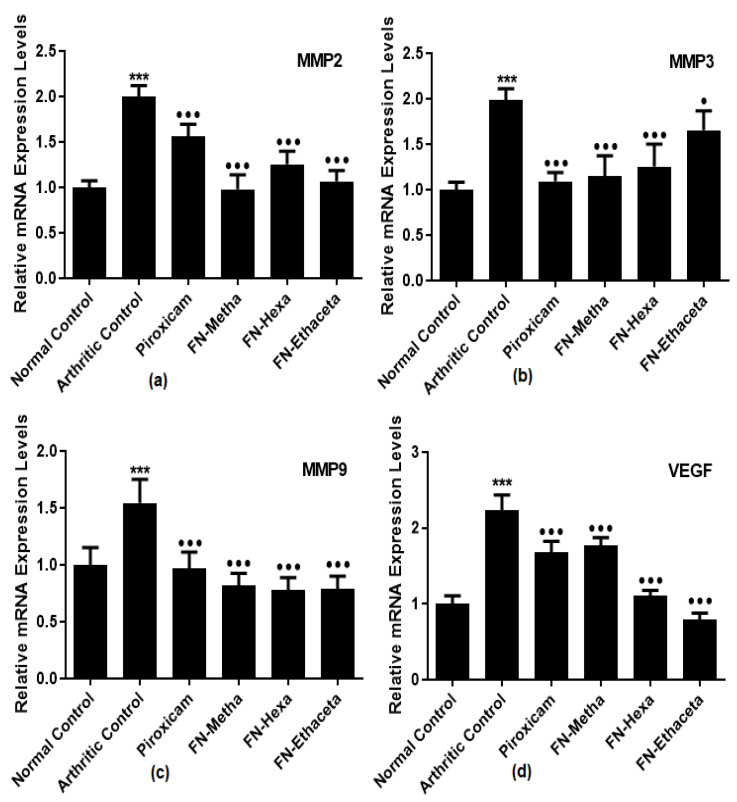
FN-Meth, FN-Hexa, and FN-Ethaceta attenuated the expression levels of (**a**) MMP2, (**b**) MMP3, (**c**) MMP9, and (**d**) VEGF as compared to the arthritic control group. Here, ^●●●^ *p* <0.001 and ^●^ *p* <0.05 as compared to the arthritic group whereas *** shows the comparison between the normal control and arthritic group.

MMP2 levels were raised in the arthritic control group (2.001 ± 0.119) as compared to the normal control group (1.000 ± 0.073). Treatment with all treated groups, such as FN-Metha (0.977 ± 0.161), FN-Hexa (1.256 ± 0.144), FN-Ethaceta (1.067 ± 0.119), and Piroxicam (1.556 ± 0.137) down-regulated the levels of MMP2 as compared to arthritic control group (Figure 5a). 

Increased MMP3 expression levels were also seen in arthritic control rats (1.983 ± 0.127) as compared to normal control rats (1.001 ± 0.082). FN-Metha (1.146 ± 0.226), FN-Hexa (1.255 ± 0.246) and FN-Ethaceta (1.656 ± 0.211) groups showed significant amelioration in the MMP3 expression levels along with Piroxicam (1.092 ± 0.097) as compared to arthritic control (Figure 5b).

Increased levels of MMP9 were also detected in the arthritic control group (1.543 ± 0.208) as compared to the normal control group (1.000 ± 0.150). Treatment with all treated groups such as FN-Metha (0.817 ± 0.107), FN-Hexa (0.780 ± 0.107), FN-Ethaceta (0.788 ± 0.112), and Piroxicam (0.970 ± 0.140) down-regulated the levels of MMP9 as compared to arthritic control group (Figure 5c). 

Gene expression levels of VEGF were significantly raised in arthritic control rats (2.614 ± 0.200) as compared to normal control rats (1.000 ± 0.105). FN-Metha (1.764 ± 0.110), FN-Hexa (1.111 ± 0.068), and FN-Ethaceta (0.791 ± 0.110) groups displayed a significant reduction in the levels of VEGF. Piroxicam also showed comparable (1.681 ± 0.143) results (Figure 5d). 

### 3.7. F. nubicola Improved Hematological Markers

**Figure 6:**
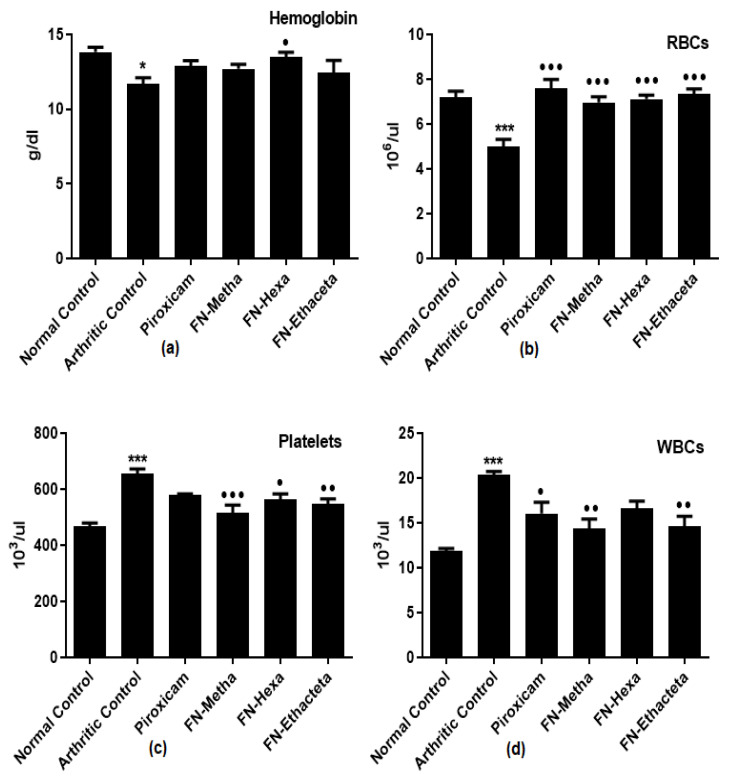
All treatment groups showed a rise of Hb content towards the value of the normal control group; however, the data showed significant elevation (**a**) in the FN-Hexa treated group only. All treated groups normalized the levels of (**b**) RBCs, whereas decreased the levels of (**c**) Platelets and (**d**) WBCs compared to the arthritic control group. Here, ^●●●^ *p* <0.001, ^●●^ *p* <0.01 and ^●^ *p* <0.05 as compared to the arthritic group, whereas *** *p* <0.001 and * *p* <0.05 as compared to the normal control group.

In arthritic control rats (11.67 ± 1.084), a reduction in hemoglobin content was detected as compared to normal control rats (13.81 ± 0.821). All treatment groups showed a rise of Hb content towards the value of the normal control group; however, the data showed marked elevation in the FN-Hexa treated group only (Figure 6a). 

In arthritic control rats (5.000 ± 0.769), a reduction in RBC levels was seen as compared to normal control rats (7.198 ± 0.656). FN-Metha (6.970 ± 0.609), FN-Hexa (7.108 ± 0.465), FN-Ethaceta (7.333 ± 0.582), and Piroxicam (7.603 ± 0.958) groups exhibited elevation in RBC levels after treatment (Figure 6b).

A prominent increase in platelets and WBCs counts was found in arthritic control rats (656.3 ± 31.79 and 20.32 ± 0.979) as compared to normal control rats (468.0 ± 21.95 and 11.89 ± 0.592), respectively. FN-Metha (515.9 ± 55.58 and 14.32 ± 2.458), FN-Hexa (564.5 ± 41.08 and 16.64 ± 1.929), and FN-Ethaceta (546.4 ± 41.07 and 14.61 ± 2.741) groups displayed reduction in the counts. The reduction is also seen in Piroxicam-treated rats (580.4 ± 6.037; and 16.03 ± 3.109) (Figure 6c,d).

### 3.8. F. nubicola Did Not Display Harm to the Liver and Kidneys

**Figure 7:**
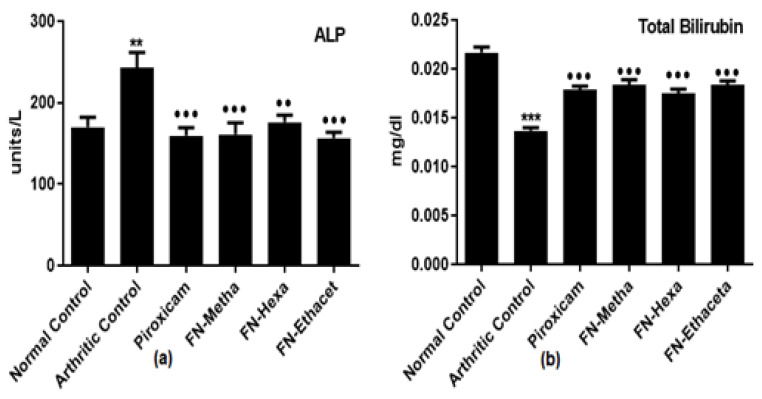
Treatment with *F. nubicola* normalized ALP levels (**a**) and improved total bilirubin levels (**b**). Here, ^●●●^ *p* < 0.001 and ^●●^ *p* < 0.01 as compared to the arthritic group, whereas *** *p* < 0.001 and ** *p* < 0.01 as compared to the normal control group.

Enhanced ALP levels (242.7 ± 46.65) were seen as compared to normal control rats (170.2 ± 29.05). FN-Metha (160.8 ± 35.04), FN-Hexa (176.2 ± 20.78), FN-Ethaceta (156.5 ± 17.47), and Piroxicam (159.3 ± 24.19) groups showed ameliorated ALP levels after treatment (Figure 7a).

In arthritic control rats (0.013 ± 0.000), a significant reduction in total bilirubin levels was seen as compared to normal control rats (0.021 ± 0.001). After treatment with FN-Metha (0.018 ± 0.001), FN-Hexa (0.017 ± 0.001), FN-Ethaceta (0.018 ± 0.001) and Piroxicam (0.017 ± 0.000), total bilirubin levels were found raised (Figure 7b).

**Figure 8:**
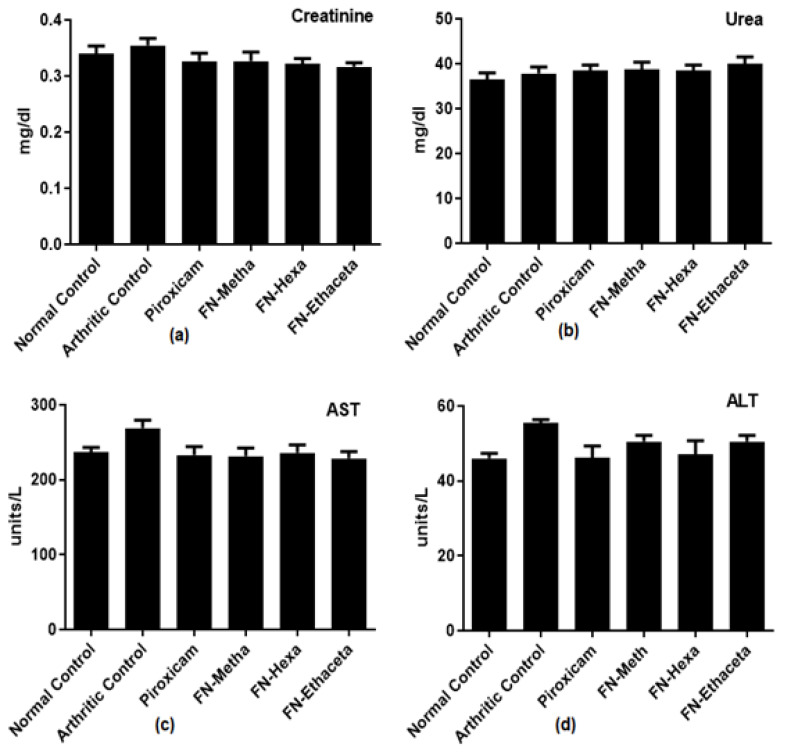
In the estimation of (**a**) Creatinine, (**b**) Urea, (**c**) AST, and (**d**) ALT levels, a significant difference was found in all groups when compared with each other.

Creatinine, Urea, AST, and ALT levels were also evaluated in the current study, and a statistically insignificant difference was detected among all groups when they were compared with each other (Figure 8).

### 3.9. GCMS Analysis of Methanol Extract, n-Hexane and Ethyl Acetate Fractions of F. nubicola

GC/MS analysis of methanol extract, n-hexane fraction, and ethyl acetate fraction of *F. nubicola* reported the presence of anti-inflammatory and antioxidant compounds. The identified constituents with their retention time, total percentage, molecular formula, molecular weight, and chemical structures are shown in Table 4 for methanol extract, Table 5 for n-hexane fraction, and Table 6 for ethyl acetate fraction.

## 4. Discussion

Rheumatoid arthritis (RA) is a heterogeneous medical condition. Generally, it is related to inflammatory changes in bones, cartilage, and synovial tissues of joints. Apart from genetic and epigenetic causes, other factors such as cigarette smoke, dust exposure, and microbiome have become the important network between the innate and adaptive immune system components [34]. NSAIDs are used as a primary therapy, but their long-term use can cause severe adverse effects related to the gastrointestinal tract and kidney. Other drug therapies like DMARDs and corticosteroids are also available, but they can cause serious side effects, too. Nowadays, researchers are considering plants as an alternate source of treatment [35].

Many studies exhibiting the anti-arthritic properties of medicinal plants have been published [14]. The ethanolic extract of *C. aurantium* Linn demonstrated anti-arthritic activity against CFA-challenged rats by declining the arthritic score, paw volume, and histological abnormalities [36]. Another study illustrated the effectiveness of various extracts of *A. solanacea* against an arthritic rat model by reducing pro-inflammatory cytokines, indicating the plant’s anti-arthritic properties [37]. The anti-arthritic efficacy of the aqueous extract of the root of *C. platypteron* at various doses was observed against different arthritic rat models [38]. The crude extracts of *P. africanum* depicted their effectiveness in CFA-Challenged rats by attenuating the production of inflammatory mediators and reactive oxygen species [39].

CFA is a commonly used model of RA induction in animals. It produces an immediate response to inflammation. This model of RA seems to produce similar effects in animals as are seen in humans [27]. The vascular exudative phenomenon observed in this study might be attributed to the flow of immune cells toward the injected area in the acute phase of inflammation. The severity of cartilage destruction and degree of synovial hyperplasia play an important role in the formation of pannus formation, which finally leads to bone destruction [34] and may be ascribed to the production of pro-inflammatory mediators in the chronic phase [28]. Bone erosion, inflammation, synovial hyperplasia, and pannus formation are the elements that contribute to the advancement of the disease [40]. In the current study, histopathological scores, paw edema, and arthritic scores were found to be reduced in treatment groups.

Pro-inflammatory cytokines, lymphocytes, monocytes, and synovial cells are the prominent stimulators in the progression of RA. Raised levels of these pro-inflammatory (TNFα, IL6, IL-1) cytokines activate pathological events leading to arthritic development [29]. TNFα is the most prominent cytokine in the synovial tissue. Mainly, activated macrophages release TNFα, but it is also produced by neutrophils and activated lymphocytes [41]. TNFα activation causes the stimulation of PGE2, IL-1β, and IL6, leading to synovial hyperplasia, production of enzymes, and collagenase activation, resulting in arthritic deterioration [27]. IL6 is an immune modulator that plays a role in the activation and prolongation of inflammatory events [42]. IL6 is released by fibroblast-like synoviocytes (FLS) and activated macrophages [41]. IL6 is responsible for the activation of the immunological responses, accumulation of auto-antibodies, and resorption of bones [27]. IL-1β is a pro-inflammatory cytokine that is released by T-cells and macrophages. It is also a powerful activator of bone resorption, like IL6. Moreover, IL-1β, along with other mediators such as TNFα and IL6, stimulate the activation of pannus formation, synovial hyperplasia, and inflammatory cell infiltration to promote bone damage [43]. The recent study showed down-regulation of pro-inflammatory cytokines such as TNFα, IL6, and IL-1β in plant-treated groups as compared with the disease group.

NF-ĸB is a cytokine-induced transcription factor and a principal regulator of many inflammatory cytokines, such as IL-1β and IL6. Primarily, it is found in the form of inactive complexes with inhibitory proteins in the cytoplasm of the cell. The TNFα and IL-1β activate the inflammatory signals and promote the deactivation of inhibitory proteins, resulting in the release of free NF-ĸB. Subsequently, the liberated NF-ĸB moves toward the nucleus and stimulates several inflammatory signals in RA [44]. High levels of PGE2 also cause bone destruction by regulating cytokine production [45]. In the current study, the decline in the levels of NF-kB and PGE2 was observed in plant-treated groups as compared with the disease group.

MMPs are the zinc-dependent endopeptidases that take part in the degradation of all extracellular matrix components [46]. Expression levels of MMPs generally tend to enhance in various inflammatory diseases [47]. Non-collagen matrix components of the joints are degraded by the elevated levels of MMP enzymes such as MMP2, MMP3, and MMP9. In RA, they are expressed via the activation of IL-1β and TNFα via many signal transduction pathways [11]. Vascular endothelial growth factor (VEGF) is identified as a significant mediator of angiogenesis, and raised angiogenesis is believed to mediate the prolongation of RA [10]. In the present study, the expression of levels of MMP2, MMP3, MMP9, and VEGF was found ameliorated in plant treated groups.

To analyze the probable hepatotoxic and nephrotoxic effects of the plant, the levels of ALP, AST, ALT, Total bilirubin, urea, and creatinine in serum were determined [48]. Increased levels of liver enzymes (ALP, AST, ALT) indicate the release of enzymes in circulation due to liver pathology [26]. ALP is known as a biochemical biomarker of bone damage. Serum ALP levels also become elevated in RA [49]. Bilirubin has powerful antioxidant and immunomodulatory properties. Previous studies suggested that bilirubin should be considered a protective biomarker for autoimmune disease. Increased levels of bilirubin activate the suppression of pro-inflammatory cytokines and inflammatory cell proliferation. A previous study showed that serum bilirubin levels were lower in RA patients than healthy controls [50]. Creatinine and urea are the biomarkers of renal disease. They are both normally filtered out from the blood by the kidney. In disease conditions, kidneys do not excrete them properly, and their levels become elevated in circulation [51]. In the current study, an insignificant difference in the levels of ALT, AST, urea, and creatinine was observed as compared with the normal control group, which suggests that no harm to the kidney and liver was performed by plant treatment in test doses, whereas in plant treated groups, a significant reduction in the ALP levels and elevation in total bilirubin levels was determined when compared with arthritic group.

Anemia is one of the noticeable features of RA [49]. In anemia, irregularities in the accumulation of iron in the reticuloendothelial system and synovial tissues, as well as bone marrow damage, are responsible for the lower levels of RBC and Hb. In RA, WBC and Platelets counts are increased due to the initiation of the immune system in response to antigens outbreak [52]. Treatment with plants nearly normalized all the hematological markers as compared with the disease group.

The GC-MS analysis showed that the anti-inflammatory activity of plant extract and fractions is due to the presence of previously reported anti-inflammatory and antioxidant phytochemicals. γ—Sitosterol is known to be an anti-inflammatory and antioxidant agent [53,54]. 9, 12, 15-Octadecatrienoicacid, methyl ester acts as an anti-arthritic and anti-inflammatory agent [55]. Vitamin E produces an anti-inflammatory effect by targeting nociception, cytokine production, and cell infiltration. Its antioxidant properties also play an essential role in its anti-inflammatory effects [56]. D-mannose works as an anti-inflammatory agent and takes part in the reduction in osteoclasts and pro-inflammatory cytokines production [57]. Betulin possesses anti-inflammatory activity comparable to dexamethasone [58]. Phytol also has strong anti-inflammatory properties [59,60,61]. Hexadecanoic acid, methyl ester, and 5-Hydroxymethylfurfural are known to be antioxidant agents [55,62].

Further research is required to standardize the plant extract before its therapeutic use. There is a need for toxicity studies in the future to determine the carcinogenic, teratogenic, and mutagenic effects of the plant. Even though GCMS analysis is used to identify the phytochemicals, in the future, there is still a need for LCMS study to identify the wide range of phytochemicals. The effects of *F. nubicola* in the different signaling transduction pathways could also be studied in the future.

## 5. Conclusions

The data revealed the treatment with *F. nubicola* declined the arthritic advancement, as evidenced by inhibition of arthritic score, paw volume, pannus formation, bone erosion, and inflammatory cell infiltration. Treatment with *F. nubicola* showed a noticeable reduction in the progression of rheumatoid arthritis and suggested the down-regulation of pro-inflammatory cytokines such as TNFα, IL6, and IL-1β, along with matrix metalloproteinases such as MMP2, MMP3, and MMP9 as the probable causes. In addition, the reduction in levels of NF-κB, VEGF, and PGE2 might also be attributed to the amelioration of rheumatoid arthritis in the current study. Future studies may aim to explore the therapeutic use of *F. nubicola* as adjunct therapy with conventional medicine; however, there is a need to perform clinical trials to determine the safety and therapeutic efficacy of the plant. It might also be suggested that the anti-inflammatory and antioxidant phytochemicals in the plant extract and fractions may be responsible for the ameliorative effects of *F. nubicola*.

## Figures and Tables

**Table 1:** *F. nubicola* reduced arthritic development.

Days	Normal Control	Arthritic Control	Piroxicam	FN-Metha	FN-Hexa	FN-Ethaceta
8th	0.0 ± 0.0	3.7 ± 0.5	3.5 ± 0.6	3.7 ± 0.5	3.3 ± 0.5	3.5 ± 0.5
13th	0.0 ± 0.0	3.7 ± 0.5	2.3 ± 0.5 ***	2.5 ± 0.5 **	2.5 ± 0.5 **	2.3 ± 0.5 ***
18th	0.0 ± 0.0	3.8 ± 0.4	2.0 ± 0.9 **	2.0 ± 0.9 **	2.2 ± 0.8 **	1.7 ± 0.8 ***
23rd	0.0 ± 0.0	3.9 ± 0.1	1.8 ± 0.9 ***	1.5 ± 0.8 ***	1.6 ± 0.4 ***	1.6 ± 0.7 ***

Values were taken as mean ± SD. When other group’s values were compared with arthritic control then ** *p* < 0.01 and *** *p* < 0.001 were considered.

**Table 2:** *F. nubicola* reduced paw volume.

Days	Normal Control	Arthritic Control	Piroxicam	FN-Metha	FN-Hexa	FN-Ethaceta
8th	0.0 ± 0.0	1.4 ± 0.02	1.4 ± 0.02	1.4 ± 0.01	1.4 ± 0.010	1.4 ± 0.01
13th	0.0 ± 0.0	1.6 ± 0.02	1.2 ± 0.01 ***	1.3 ± 0.01 ***	1.3 ± 0.01 ***	1.3 ± 0.0 ***
15th	0.0 ± 0.0	1.9 ± 0.02	1.05 ± 0.02 ***	0.9 ± 0.01 ***	1.0 ± 0.01 ***	1.0 ± 0.01 ***
23rd	0.0 ± 0.0	2.1 ± 0.10	0.71 ± 0.02 ***	0.7 ± 0.009 ***	1.0 ± 0.03 ***	0.7 ± 0.01 ***

Values were taken as mean ± SD. When other group’s values were compared with arthritic control, then *** *p* < 0.001 were considered.

**Table 3:** *F. nubicola* reduced histopathological parameters.

Parameter	Normal Control	Arthritic Control	Piroxicam	FN-Metha	FN-Hexa	FN-Ethaceta
Inflammatory cells	0.0 ± 0.0	3.7 ± 0.5	2.0 ± 0.6 ***	2.2 ± 0.4 ***	1.3 ± 0.5 ***	1.3 ± 0.8 ***
Bone Erosion	0.0 ± 0.0	3.2 ± 0.4	1.0 ± 0.6 ***	1.2 ± 0.4 ***	1.5 ± 0.5 ***	1.7 ± 0.5 ***
Pannus Formation	0.0 ± 0.0	2.8 ± 0.8	1.3 ± 0.5 **	1.5 ± 0.8 **	0.7 ± 0.5 ***	1.0 ± 0.6 ***

Values were taken as mean ± SD. When the other group’s values were compared with arthritic control, then ** *p* < 0.01 and *** *p* < 0.001 were considered. The disease was not induced in the normal control group, so the values of this group were taken as 0.

**Table 4:** List of identified components of methanol extract from mass chromatograms.

Name of Identified Compound	Molecular Formula	Molecular Weight (g/mol)	Retention Time	Total (%Age)	Structure
2,5-Furandione, dihydro-3-methylene-	C_5_H_4_O_3_	112	5.134	1.313%	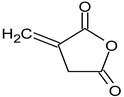
1-Hexanol,2-ethyl-	C_8_H_18_O	130	7.237	3.758	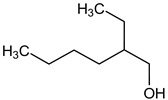
Methane, diethoxy	C_5_H_12_O_2_	104	7.757	8.919	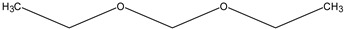
Cyclohexane,1,4-diethoxy-, trans-	C_10_H_20_O_2_	172	8.457	4.459	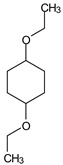
5-Hydroxymethylfurfural	C_6_H_6_O_3_	126	11.517	4.395	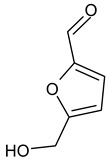
Phenol,2-propyl-	C_9_H_12_O	136	13.725	1.671	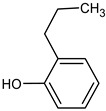
4-Tetradecene, (z)-	C_14_H_28_	196	13.906	1.39	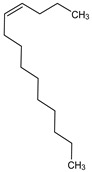
Phenol,2,4-bis (1.1-dimethylethyl)-	C_14_H_22_O	206	15.533	9.245	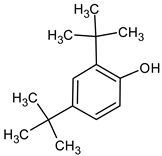
d-Mannose	C_6_H_12_O_6_	180	17.04	7.007	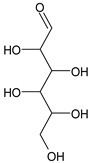
Hexadecanoic acid, methyl ester	C_17_H_34_O_2_	270	20.115	6.422	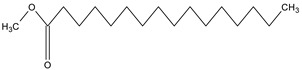
n-Hexadecanoic acid	C_16_H_32_O_2_	256	20.454	5.57	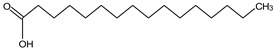
Methyl 10-trans,1 2-cis-octadecadienoate	C_19_H_34_O_2_	294	21.757	3.59	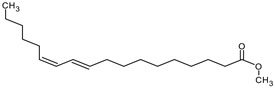
9,12,15-octadecatrienoic acid, methyl ester (Z, Z, Z)-	C_19_H_32_O_2_	292	21.818	5.636	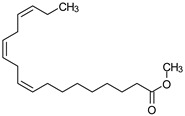
Phytol	C_20_H_40_O	296	21.923	2.674	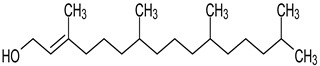
Methyl stearate	C_19_H_38_O_2_	298	22.021	3.37	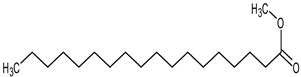
Octadecanoic acid	C_18_H_36_O_2_	284	22.33	3.696	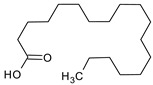
Propanoic acid,2-(3-acetoxy-4,4,14-trimethylandrost-8-en-17-yl)-	C_27_H_42_O_4_	430	24.568	3.756	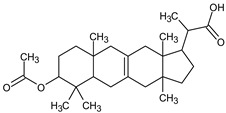
Phenol,2,2′-methylenebis 1-6-(1,1-dimethylethyl)-4-methyl-	C_23_H_32_O_2_	340	24.643	10.24	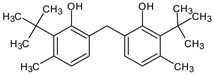
Diisooctyl phthalate	C_24_H_38_0_4_	390	25.6	4.749	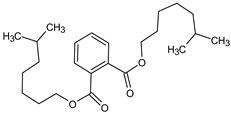
γ—sitosterol	C_29_H_50_O	414	31.516	8.14	

**Table 5:** List of identified components of n-hexane fraction from mass chromatograms.

Name of Identified Compound	Molecular Formula	Molecular Weight (g/mol)	Retention Time	Total (%Age)	Structure
2-Pyrrolidinone,1-methyl-	C_5_H_9_NO	99	7.470	3.781%	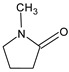
Phenol,2,4-bis (1.1-dimethylethyl)-	C_14_H_22_O	206	15.533	0.778%	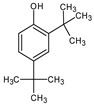
Hexadecanoic acid, methyl ester	C_17_H_34_O_2_	270	20.115	2.099%	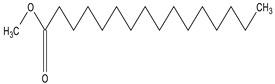
n-Hexadecanoic acid	C_16_H_32_O_2_	256	20.461	1.322%	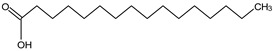
1,2-Benzenedicarboxylic acid, butyl 8-methylnonyl ester	C_22_H_34_O_4_	362	20.544	1.084%	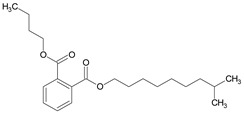
9,12-Octadecadienoic acid (Z, Z)-, methyl ester	C_19_H_34_O_2_	294	21.757	1.629%	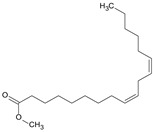
9,12,15-octadecatrienoic acid, methyl ester (Z, Z, Z)-	C_19_H_32_O_2_	292	21.818	2.110%	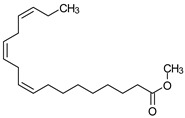
Phytol	C_20_H_40_O	296	21.923	3.246%	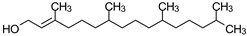
Estra-1,3,5(10)-trien-17β-ol	C_18_H_24_O	256	22.157	2.504%	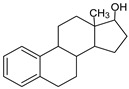
Tributyl acetyl citrate	C_20_H_34_O_8_	402	23.257	1.204%	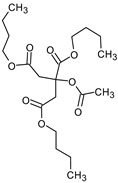
Phenol,2,2′-methylenebis 1-6-(1,1-dimethylethyl)-4-methyl-	C_23_H_32_O_2_	340	24.643	3.754%	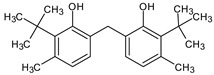
Phenol,2,2′-methylenebis [6-(1,1-dimethylethyl)-4-ethyl-	C_25_H_36_O_2_	368	25.382	1.203%	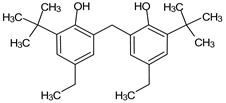
Bis(2-ethylhexyl) phthalate	C_24_H_38_O_4_	390	25.615	39.211%	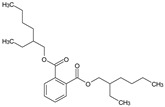
2,2,4-Trimethyl-3-(3,8,12,16-tetramethyl-hetadeca-3,7,11,15-tetraenyl)-cyclohexanol	C_30_H_52_O	428	27.635	0.802%	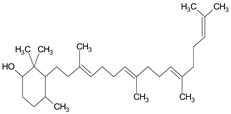
2H-1-Benzopyran-6-ol,3,4-dihydro-2,8-dimethyl-2-(4,8,12-trimethyltridecyl)-, [2R-[2R*(4R*,8R*)]	C_27_H_46_O_2_	402	28.494	1.279%	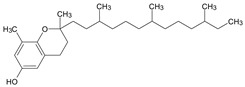
Vitamin E	C_29_H_50_O_2_	430	29.797	1.971%	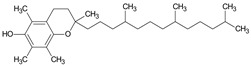
Betulin	C_30_H_50_O_2_	442	30.182	6.166%	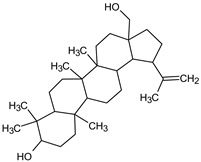
γ -Sitosterol	C_29_H_50_O	414	31.531	20.033%	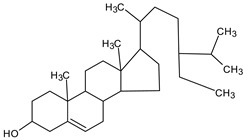
Methylenebis(2,4,6-triisopropylphenylphosphine)	C_31_H_50_P_2_	484	32.374	4.756%	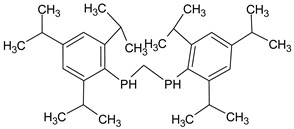
Spirost-8-en-11-one,3-hydroxy-, (3β,5α,14β,20β,22β,25R)-	C_27_H_40_O_4_	428	32.962	1.068%	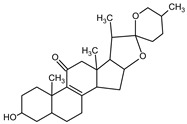

**Table 6:** List of identified components of ethyl acetate fraction from mass chromatograms.

Name of Identified Compound	Molecular Formula	Molecular Weight (g/mol)	Retention Time	Total (%Age)	Structure
Phenol,4-propyl-	C_9_H_12_O	136	13.657	2.287%	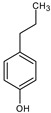
Hexadecanoic acid, methyl ester	C_17_H_34_O_2_	270	20.114	2.406%	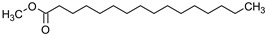
n-Hexadecanoic acid	C_16_H_32_O_2_	256	20.484	3.156%	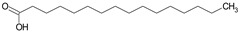
9,12-Octadecadienoic acid (Z, Z)-, methyl ester	C_19_H_34_O_2_	294	21.757	2.294%	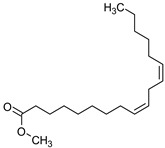
9,12,15-octadecatrienoic acid, methyl ester (Z, Z, Z)-	C_19_H_32_O_2_	292	21.825	3.195%	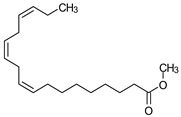
Phytol	C_20_H_40_O	296	21.923	2.206%	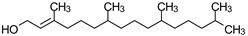
9,12-Octadecadienoic acid (Z, Z)-	C_18_H_32_O_2_	280	22.126	1.424%	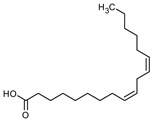
9,12-Octadecadienoic acid (Z, Z)-	C_18_H_32_O_2_	280	22.179	3.478%	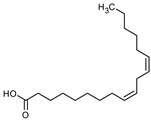
Hexanedioic acid, bis(2-ethylhexyl) ester	C_22_H_42_O_4_	370	24.387	4.085%	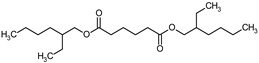
Phenol,2,2′-methylenebis 1-6-(1,1-dimethylethyl)-4-methyl-	C_23_H_32_O_2_	340	24.651	2.691%	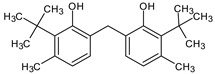
Bis(2-ethylhexyl) phthalate	C_24_H_38_O_4_	390	25.638	52.054%	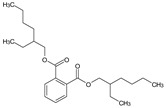
12-Methyl-E, E-2,13-octadecadien-1-ol	C_19_H_36_O	280	26.640	1.455%	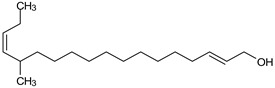
Octadecanoic acid, decyl ester	C_28_H_56_O_2_	424	26.715	1.401%	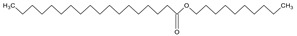
Vitamin E	C_29_H_50_O_2_	430	29.805	1.306%	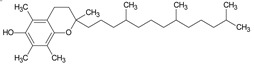
γ—Sitosterol	C_29_H_50_O	414	31.546	16.563%	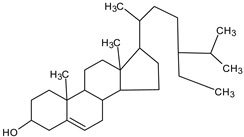

## Data Availability

Data will be provided upon reasonable request.
